# The adjuvant AlhydroGel elicits higher antibody titres than AddaVax when combined with HIV-1 subtype C gp140 from CAP256

**DOI:** 10.1371/journal.pone.0208310

**Published:** 2018-12-17

**Authors:** Michiel T. van Diepen, Rosamund Chapman, Penny L. Moore, Emmanuel Margolin, Tandile Hermanus, Lynn Morris, Phindile Ximba, Edward P. Rybicki, Anna-Lise Williamson

**Affiliations:** 1 Institute of Infectious Disease and Molecular Medicine, Faculty of Health Science, University of Cape Town, South Africa; 2 Division of Medical Virology, Department of Pathology, University of Cape Town, South Africa; 3 Center for HIV and STIs, National Institute for Communicable Diseases of the National Health Laboratory Service, Johannesburg, South Africa; 4 Faculty of Health Sciences, University of the Witwatersrand, Johannesburg, South Africa; 5 Centre for the AIDS Programme of Research in South Africa (CAPRISA), University of KwaZulu-Natal, Congella, South Africa; 6 Biopharming Research Unit, Department of Molecular and Cell Biology; University of Cape Town, South Africa; George Washington University School of Medicine and Health Sciences, UNITED STATES

## Abstract

With the HIV-1 epidemic in southern Africa still rising, a prophylactic vaccine against the region’s most prolific subtype (subtype C) would be a significant step forward. In this paper we report on the effect of 2 different adjuvants, AddaVax and AlhydroGel, formulated with HIV-1 subtype C gp140, on the development of binding and neutralising antibody titres in rabbits. AddaVax is a squalene-based oil-in-water nano-emulsion (similar to MF59) which can enhance both cellular and humoral immune responses, whilst AlhydroGel (aluminium hydroxide gel) mainly drives a Th2 response. The gp140 gene tested was derived from the superinfecting virus (SU) from participant CAP256 in the CAPRISA 002 Acute infection cohort. The furin cleavage site of the Env protein was replaced with a flexible linker and an I559P mutation introduced. Lectin affinity purified soluble Env protein was mainly trimeric as judged by molecular weight using BN-PAGE and contained intact broadly neutralising epitopes for the V3-glycan supersite (monoclonal antibodies PGT128 and PGT135), the CD4 binding site (VRC01) and the V2-glycan (PG9) but not for the trimer-specific monoclonal antibodies PG16, PGT145 and CAP256-VRC26_08. When this soluble Env protein was tested in rabbits, AlhydroGel significantly enhanced soluble Env and V1V2 binding antibodies when compared to AddaVax. Finally, AlhydroGel resulted in significantly higher neutralization titres for a subtype C Tier 1A virus (MW965.26) and increased neutralization breadth to Tier 1A and 1B viruses. However, no autologous Tier 2 neutralisation was observed. These data suggest that adjuvant selection is critical for developing a successful vaccine and AlhydroGel should be further investigated. Additional purification of trimeric native-like CAP256 Env and/or priming with DNA or MVA might enhance the induction of neutralizing antibodies and possible Tier 2 HIV-1 neutralisation.

## Introduction

Despite treatment and other interventions the HIV pandemic continues to grow, and a combination of prevention modalities needs to be implemented [1]. An effective prophylactic vaccine would have a major impact on limiting new infections, and is an essential goal in the control of HIV [2]. Design of an HIV vaccine is challenging, however, partly because there are few correlates of protection. While many vaccines have neutralising antibodies as a correlate of protection [3], the RV144 Thai phase 3 HIV vaccine trial showed that gp120 V1V2 region binding antibodies correlated with a reduced risk of HIV-1 infection by their Fc-mediated effector functions (including both antibody-dependant cellular cytotoxicity and antibody dependent cellular phagocytosis) [4–6]. In the non-human primate (NHP) model passive immunisation with neutralising antibodies has been shown to protect from SHIV challenge [7–9]. Therefore, it is also desirable for HIV vaccines to elicit a robust antibody response including neutralising antibodies [10]. The pathways to obtaining neutralising antibody responses appear to differ from those that elicit binding antibodies, as high titre binding antibodies do not correlate with neutralising antibody titres [11]. Neutralising antibody responses are associated with high levels of CD4+ T follicular helper cells (Tfh) and low levels of T regulatory cells (Treg) in the germinal centres [11, 12].

A remaining challenge in the design of HIV vaccines is to elicit neutralising antibody responses that are broad enough to prevent infection from the wide range of circulating HIV subtypes [13]. Selection and design of the antigen is an important component of vaccine strategy [14, 15]. For the present study, an HIV envelope sequence was selected from a person who had developed broadly neutralising antibodies (bNAbs). The sequence used for the production of HIV subtype C soluble Env protein was based on a virus isolated from a patient in the South African CAPRISA 002 acute infection cohort, patient CAP256, who developed bNAbs following a secondary infection of HIV-1 approximately 15 weeks after the primary infection [16]. The CAP256 superinfecting viral env (CAP256 SU) was selected as it elicited bNAbs in this donor [17] and is sensitive to several prototype broadly neutralising monoclonal antibodies [18]. In addition, the enhanced reactivity of this Env for certain bNAb precursors makes it an appealing candidate immunogen [19, 20].

One of the goals of an Env based HIV vaccine is to induce Tier 2 neutralising antibodies which would be able to neutralise circulating infectious virus. To date none of the vaccines in clinical trial have induced such a response [21, 22] and it is thought that this is due to Env not being correctly presented as a native trimer. The native trimer consists of three gp120 and three gp41 subunits linked non co-valently that arise from a furin cleaved gp160 precursor [23]. Improved design of Env trimer to resemble native trimers include a disulfide bond between gp120 and the ectodomain of gp41, as well as other stabilizing mutations (SOSIP Env). Such SOSIP trimers based on gp140 have antigenic characteristics similar to native Env trimers [23] and the BG505 SOSIP trimer has been demonstrated to induce Tier 2 neutralising antibodies in animal models [24]. Not all Env antigens engineered as SOSIP trimers form stable structures resembling the native soluble [23]. In addition, SOSIP based Envs have to be fully cleaved by furin which can complicate the production of the vaccine [25]. Another approach to improving the design of Env is to replace the native gp120-41 furin cleavage site with a native flexible linker (NFL) to create uncleaved but well-ordered trimers [25]. In a comparison of BG505 SOSIP and NFL based vaccines in NHPs it was shown that similar end-point neutralising antibody responses were induced but the NFL trimers response were slower [24]. In this paper the flexible linker modification was used on CAP256 SU.

Subunit vaccines tend to be poorly immunogenic, and their immunogenicity can be enhanced substantially by formulation with the appropriate adjuvant [26]. Adjuvant selection is an important factor in promoting the desirable cellular and/or humoral immune response to be elicited by a vaccine. In an NHP model, a vaccination regimen comprising ALVAC-simian immunodeficiency virus (SIV) + gp120 provided protection from SIVmac251 acquisition only when alum and not when MF59 was used as the adjuvant [27]. This is an important result as many of the ongoing clinical trials of candidate HIV vaccines are using similar adjuvants with the protein boosts [28]. In this paper we report on the effect of 2 different adjuvants (AddaVax and AlhydroGel) formulated with HIV-1 subtype C gp140 on the development of binding and neutralising antibody titres in rabbits. AddaVax is a squalene-based oil-in-water nano-emulsion similar to MF59. These types of adjuvants are thought to enhance both cellular and humoral immune responses [29, 30]. AlhydroGel (aluminium hydroxide gel) on the other hand mainly drives a Th2 response [31].

When evaluated for activity with influenza vaccines, AddaVax-adjuvanted formulations were more immunogenic than aluminium hydroxide gel–adjuvanted formulations, driving significantly higher humoral immune response against influenza virus A H7N9 virus, and strong cross-reactive responses including neutralisation responses against a H7N7 virus [32]. Both AddaVax and AlhydroGel have been reported to keep Env (native-like trimeric) structure intact after adjuvanting [33] making a comparison easier to interpret.

## Materials and methods

### CAP256 SU V1V2 scaffolds, antibodies, plasmids, cell lines, media and reagents

The sequence of CAP256 SU gp160 (clone CAP256.206sp.032.C9) has been previously described (GenBank: KF241776.1) [18]. CAP256 SU V1V2 scaffolded protein was produced as follows, V1V2 scaffolded mini-proteins with a His-tag were expressed in HEK293S GnTI^-^ cells (ATCC CRL-3022) grown at 37°C, 5% CO2, 70% humidity, 125 rpm, to yield a uniform low-mannose glycan content. Cultures were harvested after seven days by centrifugation at 3000g and purified by sequential Ni-NTA and size exclusion (SEC) chromatography. Antigenicity and specificity was confirmed by ELISA using V2-specific mAbs (PG9, CAP256-VRC26.25) as positive controls and CD4bs mAbs (VRC01) as a negative control [34]. Soluble, trimeric BG505_664-His was purified using lectin affinity chromatography followed by size exclusion chromatography. The trimeric fraction was then purified further by negative selection using antibody 447-52D. Anti-Env human monoclonal antibodies PG9, PG16, PGT128, PGT135, PGT145, CAP256-VRC26_08 VRC01, F015 and 447-52D were expressed in FreeStyle 293F cells (Life Technologies) using the PEIMAX transfection reagent (Polysciences). Monoclonal antibodies were purified from cell-free supernatants after 6 days using protein A affinity chromatography [17]. Goat anti-gp160 (408/5104) was obtained from MRC ADP (UK). The mammalian expression plasmid pTHCapR was from the lab collection (BRU, UCT, Cape Town, SA) [35]. HEK293 and HEK293T cells were grown in DMEM High Glucose + L-Glutamine (Lonza, Basel) + 10% FCS (Biochrom, Holliston) + 1x Pen/Strep (Thermo Fisher Scientific, Waltham).

### CAP256 SU GP140-FL-IP cloning, expression and isolation

The human tissue plasminogen activator (TPA) leader sequence was human codon optimised, synthesised by GenScript (Nanjing) and cloned into pTHCapR to increase target sequence secretion efficiency. This plasmid was renamed pMExT for Mammalian Expression with TPA leader.

**Fig 1:**
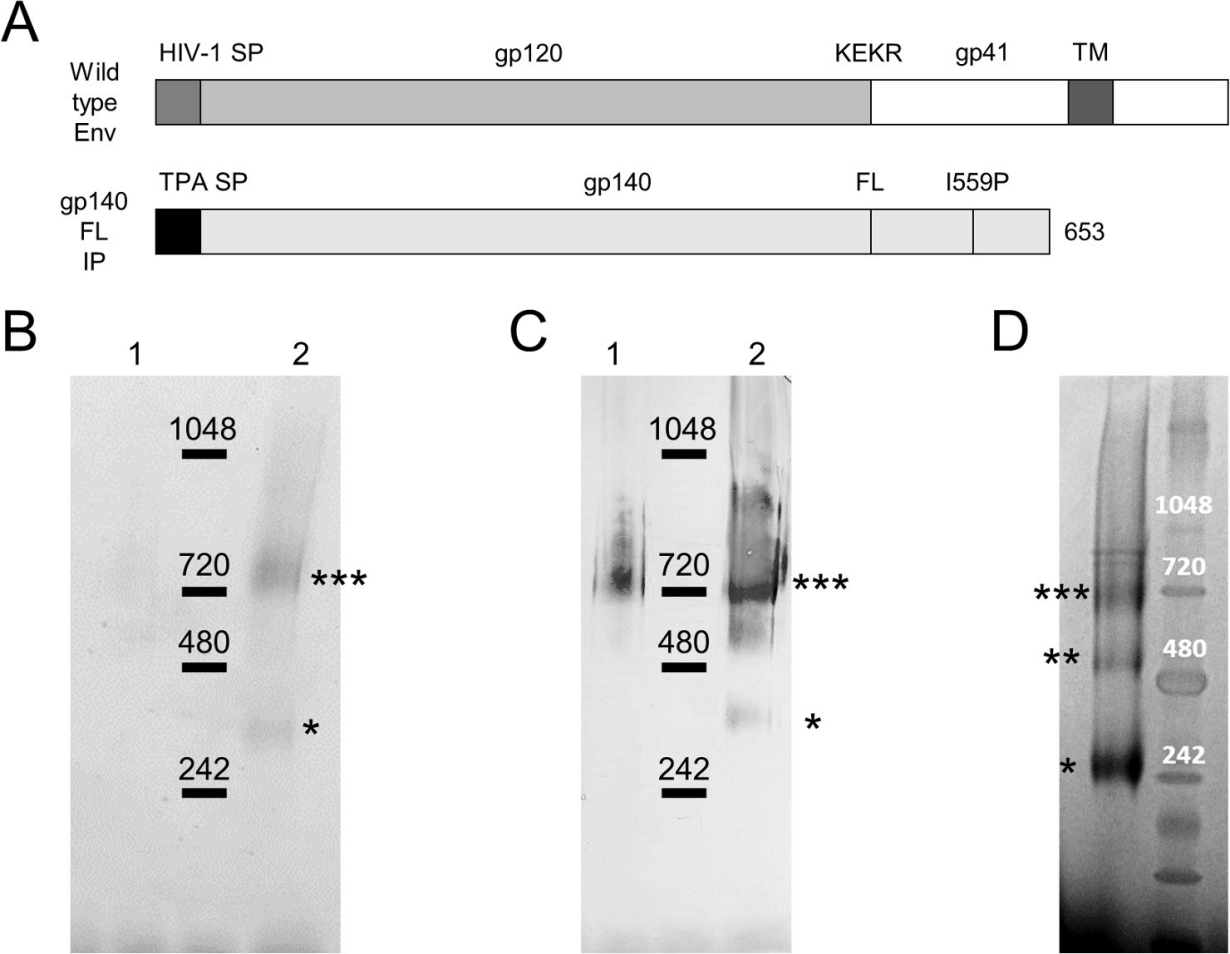
CAP256 gp140-FL-IP protein vaccine design and characterisation. (A) Schematic representation of wild type Env and truncated CAP256 gp140-FL-IP (soluble CAP256 Env) in which the native signal peptide is replaced with the human tissue plasminogen activator (TPA) sequence, the furin cleavage site with a flexible linker (FL) sequence and an I559P mutation is introduced. A protein band around 720 kDA, the molecular weight of Env trimers, was observed by Coomassie staining (B) and anti-Env western blotting (C) of NativePAGE protein gels in both the eluate (lane 1) and concentrated eluate (lane 2) after lectin purification (indicated with ***) of CAP256 gp140-FL-IP. Some Env monomers, as defined by molecular weight (MW), were observed in the concentrated eluate (*). (D) The protein profile of CAP256 gp140-FL-IP-His differed when analysed by Coomassie stained NativePAGE protein gels with a much larger proportion of Env MW monomers compared to MW trimers and the appearance of Env MW dimers (indicated with **). Molecular weight was estimated using NativeMark Unstained Protein Standard.

The CAP256 SU gp160 sequence was modified as follows (Fig 1A): the native leader sequence was removed, the furin cleavage site was replaced with a flexible linker sequence (FL) [25], and an I548P mutation equivalent to the I559P in the SOSIP trimers was introduced to promote trimerisation of gp41 [36]. Finally, the transmembrane domain was removed by truncation to gp140. Recovery and purification is improved of this resulting secreted protein. The Env sequence was human codon optimised, synthesised by GenScript (Nanjing) and cloned in frame with TPA leader in pMExT thus creating pMExT Gp140-FL-IP. For structural characterization of Gp140-FL-IP, a 6x His-tag was introduced before the STOP-codon resulting in pMExT Gp140-FL-IP-His.

Stable cell lines were generated by introducing an IRES-Neomycin resistance cassette directly downstream of gp140-FL-IP or Gp140-FL-IP-His in pMExT. These constructs were transfected into HEK293 cells and passaged at least 10 times in medium containing 600 μg/ml Geneticin (Thermo Fisher Scientific, Waltham).

Gp140-FL-IP or Gp140-FL-IP-His protein was either isolated from gp140-FL-IP stably transfected cell lines (>P10) or from transiently transfected HEK293T cells that had been switched to serum-free conditions. Transient transfection was performed with polyethyleneimine (PEI, Sigma, St Louis) on the same day when cells were transferred to serum-free conditions (DMEM, Lonza, Basel). Agarose conjugated lectin (*Galanthus nivalis*, Sigma, St Louis) affinity purification columns were used to purify CAP256 gp140-FL-IP or Gp140-FL-IP-His protein from supernatant four days after changing to serum-free conditions. Columns were first washed with PBS+0.5M NaCl, followed by PBS only. Protein was eluted in PBS + 1M methyl α-D-manno-pyranoside (Sigma, St Louis) and subsequently concentrated and buffer exchanged into PBS using Vivaspin 20 MWCO 30 000 columns (GE Healthcare, Chicago). Protein was aliquoted and stored at -80°C for downstream usage. Protein concentration was determined using the *DC* Protein Assay (Bio-Rad, Hercules) against a BSA standard.

### CAP256 SU GP140-FL-IP protein characterisation

To characterise CAP256 gp140-FL-IP or Gp140-FL-IP-His, precast NativePAGE Novex 3–12% Bis-Tris Protein Gels (Thermo Fisher Scientific, Waltham) were run. Gels were either stained with Bio-Safe Coomassie (Bio-Rad, Hercules) or blotted onto PVDF membrane (Bio-Rad, Hercules). For the latter, blots were incubated with goat anti-gp160 (1:1000), followed by monoclonal anti-goat/sheep IgG–AP (1:10000)(Sigma) and detected with BCIP/NBT Phosphatase Substrate (KPL, Milford). NativeMark Unstained Protein Standard (Thermo Fisher Scientific, Waltham) for native gel electrophoresis was used for estimating molecular weight.

To assess the gp140 antigenic structure, Ni-NTA HisSorb Plates (Qiagen, Hilden) were coated for two hours with 200 ng/well CAP256 SU GP140-FL-IP-His protein from three separate isolations (*n* = 3) at room temperature. ELISA plates were washed with PBS (Lonza, Basel) and blocked using 5% non-fat milk (Sigma, St Louis) in PBS. Serial dilutions were made of anti-Env human monoclonal antibodies PG9, PG16, CAP256-VRC26_08, PGT128, PGT135, PGT145, VRC01, F105 and 447-52D in PBS + 5% non-fat milk, starting at 10 mg/ml and added to gp140 coated plates and incubated overnight at 4°C. Plates were washed with PBS and incubated with anti-human IgG HRP (1:5000) (Dako, Santa Clara) in block for one hour at room temperature. After washing with PBS, TMB ELISA Substrate (Abcam, Cambridge) was added for detection and the reaction was stopped after 10 minutes with 1N H_2_SO_4_. ELISA signal was analysed using a VersaMax ELISA Microplate Reader (Molecular Devices, Sunnyvale), which subtracted absorbance at 540 nm from 450 nm. Data points from the three separate protein isolations were averaged and fitted to a Four Parameter Logistic Regression curve (4PL curve) in GraphPad Prism 5.0.

### Rabbit immunisations

The animals used in this study were housed and maintained in accordance with the South African national guidelines for Use of Animals for Scientific Purposes (SANS Code 10386) which is also in line with EU Directive 2010/63/EU. Female New Zealand white rabbits (2.2kg weight or greater) were housed in groups of six, in open pens at the Research Animal Facility in the Faculty of Health Sciences at the University of Cape Town (UCT). The pens contained wood shavings litter, a nest box, resting shelf and pine gnawing blocks. The rabbits were fed rabbit pellets, fresh vegetables and lucerne and water was provided ad lib. All the animal procedures were approved by the UCT Animal Research Ethics Committee (reference UCT AEC 14–030) and performed by a trained animal technologist. Rabbits were randomly assigned to three groups of five rabbits to compare formulations of gp140 with squalene oil-in-water emulsion AddaVax and with AlhydroGel (both InvivoGen, San Diego). Rabbits were immunized intramuscularly in the hind leg with 42–45μg of CAP256 SU gp140-FL-IP protein, formulated in 500 μl, at weeks 0, 4, 12 and 20. The protein suspension was mixed 1:1 (*v*/*v*) with both adjuvants. All animals were bled two months before the first immunisation to obtain pre-bleed sera and were bled every four weeks once protein inoculations had started. Animals were sacrificed at week 24. The method of anaesthesia used during this study was Acepromazine (ACP) and animals were monitored during treatment for respiratory rate, body temperature, heart rate and absence of pedal reflex. For the final collection of blood at the end of the experiment the rabbits received an intravenous injection of ACP, followed by a mixture of ketamine and xylazine. The animals were then exsanguinated. The rabbits were monitored daily for any signs of pain, discomfort or stress and were weighed weekly.

### Rabbit sera characterisation

#### Binding antibody assay

To assess Env binding antibody titres in rabbit sera, ELISA experiments were performed with the following permutations. Plates were coated overnight with 10 ng/well gp140-FL-IP protein. Rabbit sera were used in the primary incubation in a serial dilution range starting at 1:10. PBST (PBS containing 0.1% Tween 20 (Sigma, St Louis) was used instead of PBS for all subsequent steps. Detection antibody was goat anti-rabbit IgG-HRP conjugate (1:10000) (Roche, Basel). ELISAs for the whole time course and all groups were performed at the same time on duplicate plates. Antibody end-point titres were calculated from 4PL curves of duplicate data points with the threshold set as twice the geometric mean of the ELISA signal over the whole, matching pre-bleed serial dilution range. Data were plotted as mean +/- SEM for whole group. Binding ELISAs to CAP256 SU V1V2 loop scaffold for week 0 and 22 were performed in a similar fashion but wells were coated with 500 ng/well of protein.

#### Neutralisation assays

Rabbit sera from week 8, 16, 22 and 24 were tested for their ability to inhibit Env-pseudotyped virions from entering a reporter cell line. Neutralisation was measured as a reduction in luciferase gene expression after a single round of infection of JC53bl-13 cells, also known as TZM-bl cells (NIH AIDS Research and Reference Reagent Program), with Env-pseudotyped viruses (Env from HIV-1 isolates MW965.26, MN.3, 6644, CA146, 1107356, CAP37, CT349, Du156, 188146, CAP256.SU, ZM53, Ce1086). Titre was calculated as the reciprocal plasma/serum dilution resulting in a 50% reduction of relative luciferase units (ID_50_). Murine leukemia virus (MuLV) was used as a negative control to determine non-specific inhibition/background.

### Statistical analysis

All statistical analysis was performed using GraphPad Prism 5.0. Two-way ANOVA with Bonferroni *post*-*hoc* testing was performed to compare serum end-point titres time courses. Serum neutralisation data was analysed using Two-way ANOVA with Bonferroni *post*-*hoc* testing for time courses and One-way ANOVA with Tukey *post*-*hoc* testing for week 22 Tier 1B data. Where no neutralisation was observed (titre <20), a value of 19 was used.

## Results

### Selection, purification and characterisation of CAP256 modified gp140

CAP256 SU Env was modified to try to generate trimeric proteins and improve the structure and antigenicity (Fig 1A). Truncation at amino acid 653 removed the transmembrane domain of gp160, in order to render the resulting gp140 protein soluble. As the native leader of Env results in poor trafficking within mammalian cells, this was replaced with the human tissue plasminogen activator (TPA) leader sequence to enhance secretion of gp140. To circumvent impaired furin processing which could lead to misfolding [37, 38], the furin cleavage site was replaced with a flexible linker (FL) sequence: GGGGS GGGGS to stabilize linkage of gp120 to gp41 [25, 39]. Finally, the I559P mutation was introduced to stabilise the gp41 trimer [36] thus generating CAP256 SU gp140-FL-IP (afterwards referred to as CAP256 gp140). A similar version containing a C-terminal His-tag was generated in parallel: CAP256 gp140-FL-IP-His.

CAP256 gp140 was purified from HEK293 cell supernatant using lectin (*Galanthus nivalis*) affinity chromatography as described in the material and methods above. The resulting protein was devoid of major impurities and mainly consisted of trimers as judged by the molecular weight (MW) following Coomassie staining and western blotting of NativePAGE protein gels (Fig 1B and 1C). However, some MW monomeric protein and high MW aggregates could be detected in the concentrated gp140 fraction. Interestingly, the profile of CAP256 gp140-FL-IP-His on NativePAGE protein gels differed quite significantly with the main species being monomeric Env, followed by trimeric Env and also some Env dimers as judged by MW (Fig 1D).

**Fig 2:**
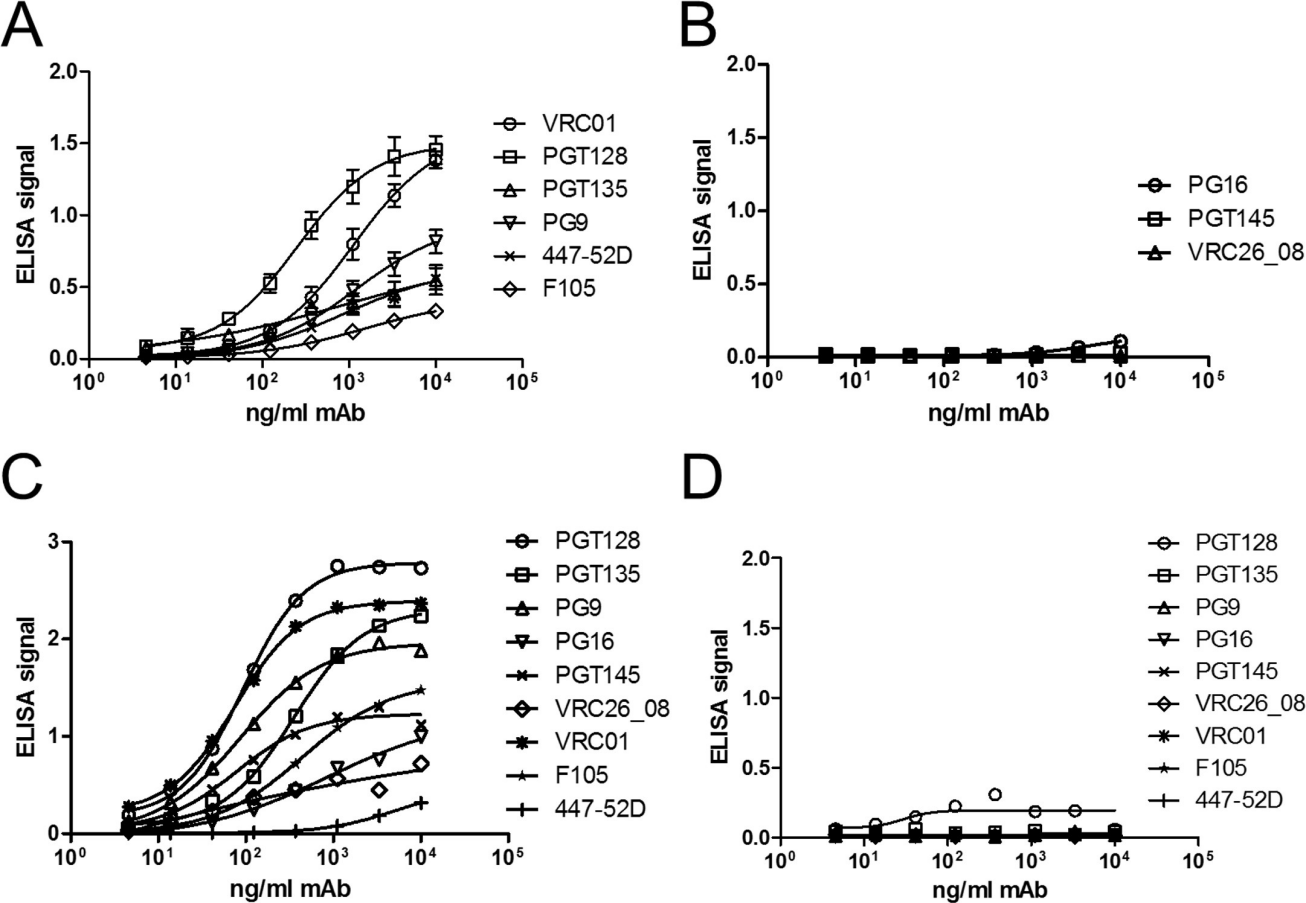
CAP256 gp140-FL-IP-His protein α-Env ELISA. Binding ELISAs using anti-Env human monoclonal antibodies to CAP256 gp140-FL-IP-His. (A) PGT128 and PGT135 binding indicate the presence of the Env V3-glycan supersite. Similarly, the CD4 binding site using VRC01 The V2-glycan epitope was detected with PG9 (A), but no binding was observed for the native-like trimer-specific MAbs PG16, PGT145 and (CAP256) VRC26_08 (B). In line with this, clear ELISA signals were observed for F105 and 446-52D, indicative of the presence of misfolded Env (A). (C) Control soluble, trimeric BG505_664-His Env protein performed as expected, with ELISA signals for native-like trimer-specific MAbs PG16, PGT145 and (CAP256) VRC26_08. (D) No binding of MAbs was observed for the no protein (PBS) control.

Further characterisation by His-capture ELISAs of CAP256 gp140-FL-IP-His using MAbs revealed that the Env V3-glycan supersite of vulnerability [40] was intact, (Fig 2A; PGT128 and PGT135) as was the CD4 binding site (VRC01). The V2-glycan MAb PG9, which binds both monomeric and trimeric Env, also bound the CAP256-SU gp140. However, native-like trimer-specfic MAbs PG16 and PGT145, both of which neutralize the CAP256 SU virus [18] failed to bind (Fig 2B). Similarly, CAP256-VRC26_08 MAb which potently neutralizes the parent virus failed to bind the gp140, suggesting that the trimer may not be adopting a native-like structure. This is further emphasized by binding of F105 and 447-52D which are MAbs being used to identify misfolded Env protein. Soluble, trimeric BG505_664-His Env protein was used to verify the His-capture ELISA and good ELISA signals were observed for all MAbs apart from 447-52D, which was used for negative selection to generate this particular control (Fig 2C). No binding was observed for any of the MAbs for the no protein (PBS) control (Fig 2D).

However, as CAP256 gp140 produced in this fashion suggested a preferentially trimeric conformation as based on molecular weight of the protein and included some of the key epitopes for the induction of neutralising antibodies, it was judged to be a suitable immunogen to compare the ability of adjuvants to elicit high titre antibodies and possibly a neutralising response in rabbits.

### Rabbit immunisation and serum anti-Env antibody characterisation

**Fig 3:**
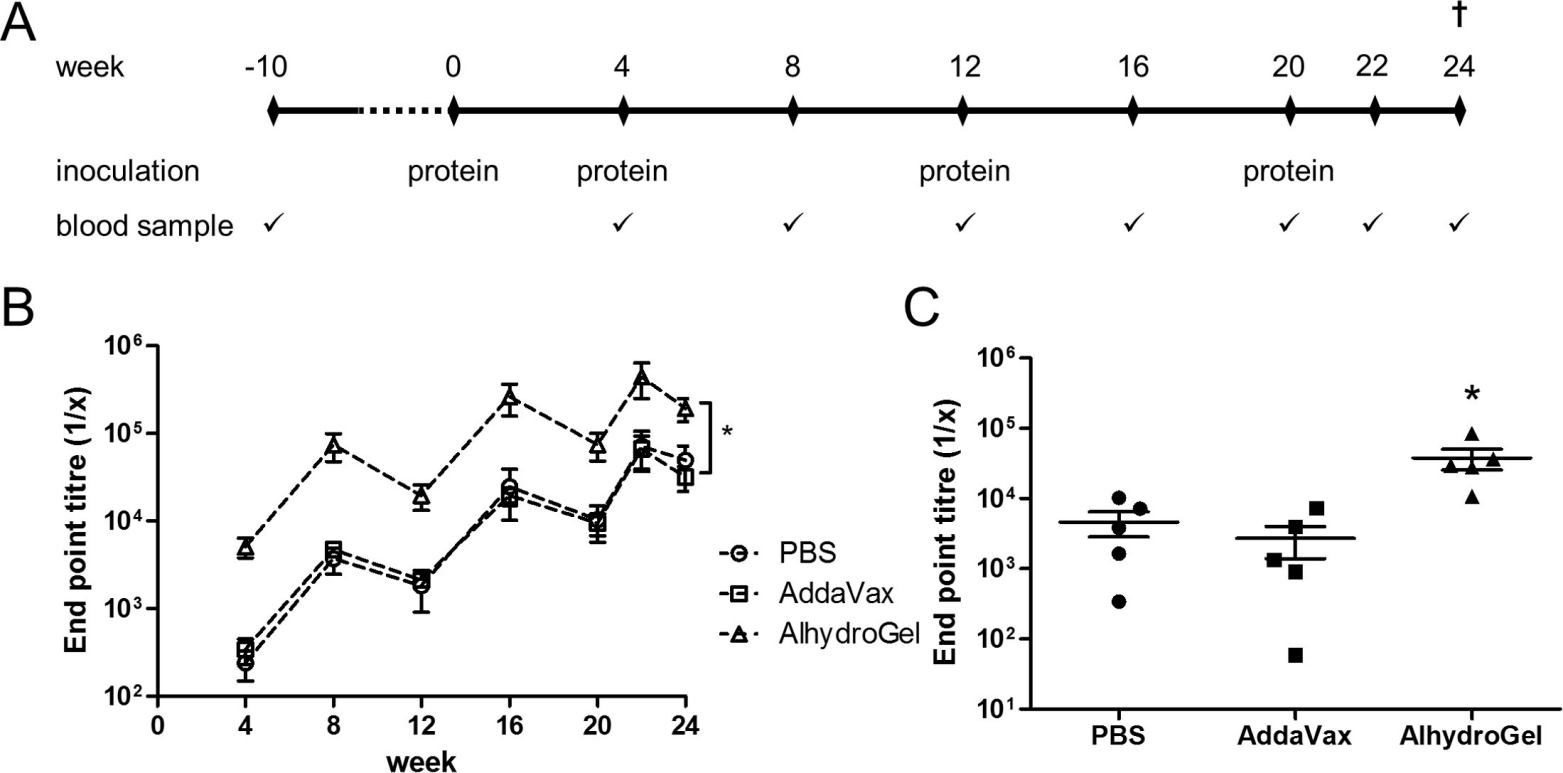
Rabbit immunisation protocol and serum characterisation. (A) Immunisation regimen. (B) CAP256 gp140 binding ELISA. Animals inoculated with adjuvant AlhydroGel had significantly higher serum anti-Env antibodies titres over the whole time course as compared to control group and AddaVax as adjuvant (p<0.0001). (C) CAP256 V1V2 loop scaffold binding ELISA week 22 sera. Animals inoculated with adjuvant AlhydroGel showed significantly higher binding to CAP256 V1V2 loop scaffold as compared to control group and AddaVax as adjuvant (p<0.05). Signal from week 0 was subtracted from week 22.

To investigate the effect of different adjuvants on the development of HIV-1 Env binding antibody titres and neutralising antibodies, gp140 was administered to rabbits intramuscularly in two different adjuvants and compared to no adjuvant PBS control (Fig 3A). An autologous gp140 binding assay was established to test for anti-Env antibody titres in rabbit sera at the different time points. Antibody end point titres for the AddaVax group were comparable to the control group for all time points (Fig 3B and S1 Table). However, Alhydrogel elicited significantly higher end point titres over the whole time course compared to both the AddaVax and control groups (p<0.0001) (Fig 3B), with titres peaking by week 22 at a dilution of 1 in 443,199 +/-192,842 for the Alhydrogel group and at 1 in 71,398 +/- 34,647 and 1 in 66,196 +/- 27,191 for PBS and AddaVax respectively.

To date, the one of the main correlates in the sole HIV-1 vaccine trial showing protection (RV144) is antibody binding to the V1V2 loop of gp160 [41]. To investigate whether our gp140 could elicit antibodies targeting this region, a binding ELISA was performed against the autologous CAP256 SU V1V2 loop which was presented on a scaffold to retain the natively folded state [34]. Good binding titres were observed for week 22 sera of all groups indicating that V2 binding antibodies were elicited by CAP256 gp140 (Fig 3C and S2 Table). As with the matching Env binding ELISA, end-point titres for the Alhydrogel group were significantly higher than the other 2 groups (p<0.05).

### Neutralisation responses after immunization

**Fig 4:**
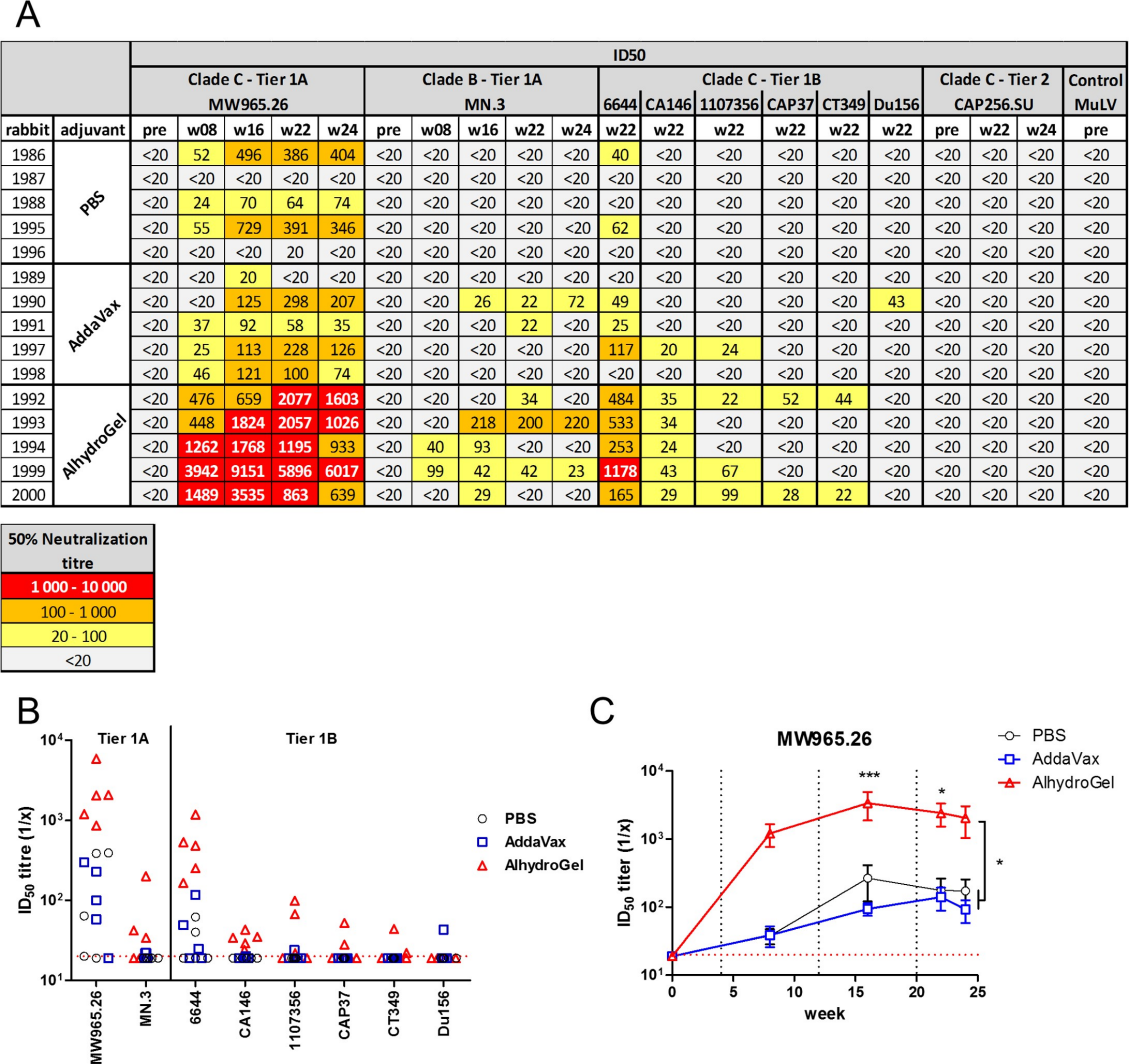
Serum neutralisation measured by the TZM-bl assay. A) The 50% neutralisation titres are color-coded to reflect their potency range as indicated. Titres below 20 are considered negative and not color-coded. B) ID_50_ titres at week 22 reflecting enhanced neutralisation in the Alhydrogel group. C) Time course of ID_50_ titres for MW965.26 are significantly higher in the Alhydrogel group. Dotted red line represent assay detection limit (1/20 dilution), all data points below detection limit are plotted as 19. Dotted black lines represent time points of protein boosts.

Similar to the ELISA assays, sera from the group receiving the adjuvant AlhydroGel performed better in inhibiting cell entry of Env-pseudotyped virions (Fig 4). Over the whole time course, the serum dilution resulting in a 50% decrease in cell entry (ID_50_) of Tier 1A Clade C virions (MW965.26) was significantly higher for the AlhydroGel group than for the control or AddaVax groups (p<0.05)(Fig 4B), with titres at week 16 and 22 significantly higher (p<0.001 and P<0.05 respectively). A similar trend was apparent for Tier 1A Clade B (MN.3); however, this was not significant. With regard to responses against Tier 1B Clade C (6644, CA146, 1107356, CAP37, CT349), neutralisation was enhanced and more animals responded in the AlhydroGel group, with neutralisation of 6644 and CA146 being significantly higher compared to the control or AddaVax groups. No neutralisation of Tier 1B Clade C virus 188146 was observed. Furthermore, none of the sera was able to neutralise the vaccine-matched Tier 2 Clade C virus (CAP256 SU) at any time point for any of the groups. Similarly, none of the sera at week 22 or 24 was capable of neutralising Tier 2 Clade C virions from isolate ZM53 or Ce1086.

## Discussion

The structure of Env is important in vaccine studies [23]: accordingly, the antigen in this study was stabilised by replacing the furin cleavage site with a flexible glycine-rich linker [37, 38], and a I559P mutation [36]. The purified CAP256 SU gp140 preparation contained trimeric species, based on molecular weight, as confirmed by BN-PAGE gel and western blot analyses, and several broadly neutralizing antibody epitopes were detected with monoclonal antibodies for CAP256 SU gp140-FL-IP-His. The V3-glycan supersite was confirmed with PGT128 and PGT135, with VRC01 confirming the CD4 binding site. Although the V2-glycan epitope was detected with PG9, the native-like trimer specific MAbs PG16 and PGT145 failed to bind. Furthermore, the isolate matched neutralising antibody MAb CAP256-VRC26_08, which potently neutralises the parent virus, failed to bind. The detection of mainly non-trimer specific neutralizing monoclonal antibody epitopes is likely due to the nature of the lectin purified Env which contains both monomeric and trimeric Env as detected by molecular weight on BN-PAGE gel. Furthermore, in the binding ELISA for these MAbs instead of CAP256 SU gp140, a His-tagged version was used which was shown to have a different profile of Env species as judged by molecular weight on BN-PAGE gels with a larger proportion of monomeric Env and Env dimers with the latter not present in CAP256 SU gp140. This might suggest that CAP256 SU gp140, which was used as the protein vaccine in the rabbit experiment, might have improved neutralizing monoclonal antibody epitopes.

We studied the kinetics of the immune response to CAP256 gp140 in rabbits immunised with no adjuvant, and the adjuvants AlhydroGel or AddaVax. All three formulations elicited an antibody response after one immunisation, and titres continued to increase until week 22 after 4 immunisations. There was however a drop in binding antibody titres at weeks 12, 20 and 24. This relatively rapid drop in binding titres to HIV Env is typical of soluble immunogens and has been seen in many studies [42]. The RV144 clinical study showed a drop in antibody titres and this was correlated with a loss in protection from HIV infection [5, 43]. The short-lived antibody response to Env is one of the challenges in HIV vaccine research, and a number of strategies to improve the longevity are being investigated. These include more immunisations, and different prime/boost combinations [22, 43, 44].

The rabbits receiving either no adjuvant or AddaVax had similar binding antibody responses, which were significantly lower than the rabbits receiving the alum-based AlhydroGel formulation. This result was unexpected, because in other studies AddaVax outperformed AlhydroGel or other alum formulations as an adjuvant [32, 45]. However, in a nonhuman primate study of ALVAC-SIV plus gp120 administered with either alum or MF59, animals receiving MF59 had stronger systemic and mucosal responses to Env than the group receiving alum [27]. Interestingly however, in this study only the alum formulated vaccine gave protection suggesting qualitative differences in antibody responses [27]. However, a comparison of these studies is complicated by differences in animal models (rabbit, mice, nonhuman primates), Env sequences (isolates and/or gp120, gp140) or virus (HIV-1, influenza).

Animals immunised with gp140 plus AlhydroGel performed significantly better in binding to the autologous CAP256 SU V1V2 loop scaffold as compared to the control group and the AddaVax group (p<0.05) further suggesting that some of the responses were V2-directed. However, more extensive epitope mapping is required to fully understand the antibody specificities in these sera and whether or not there are qualitative or simply quantitative differences between the 2 adjuvanted groups.

In summary, this study has demonstrated that the CAP256 SU Env-derived gp140 we constructed contained trimeric species (based on molecular weight) and that it was immunogenic in rabbits and could elicit HIV-1 Tier 1 neutralising antibodies. Moreover, we showed that an alum-based adjuvant—AlhydroGel—was significantly better at eliciting Tier 1 neutralising antibodies with this antigen than AddaVax, a MF59 analogue often used with Env-based antigens. The improved neutralisation titres with AlhydroGel-adjuvanted trimeric gp140 implies that this adjuvant should be used in future experiments. Although this suggestion is based on data without Tier 2 neutralization, it should be emphasized that so far Tier 2 neutralisation has only been reported for a small minority of Env sequences tested (BG505, JFRL, B41, 16055, AMC008) and the main reason for the use of a Tier 1B panel was to be able to differentiate the immunogenic properties for other Env sequences.

Further purification of the Env should be implemented to remove non-trimeric Env species which may be immunodominant or elicit off-target responses [46] to determine if Tier 2 neutralising antibodies can be induced. In addition, heterologous prime/boost strategies should be investigated to determine whether a vaccine that can induce functional binding antibodies (ADCC), bNAbs and broad, high magnitude effective, cytotoxic CD8 T cells can be generated. A recent study [47] showed that priming with chimpanzee adenovirus and non-replicating poxvirus modified vaccinia virus Ankara expressing BG505 SOSIP.664 Env induced similar Tier 2 neutralisation to BG505 SOSIP.664 Env trimers alone. Both viral vectors used in this study have also previously been shown to induce robust CD4 and CD8 T cell responses and could thus be suitable candidates for a heterologous prime [48, 49].

## Supporting information

S1 Table CAP256 gp140 binding ELISA end point titres of individual rabbits. (PDF)

S2 Table CAP256 V1V2 loop scaffold binding ELISA end point titres of individual rabbits. (PDF)

S1 Checklist NC3Rs arrive guidelines checklist. (PDF)
