# Supplementary material for: The adjuvant AlhydroGel elicits higher antibody titres than AddaVax when combined with HIV-1 subtype C gp140 from CAP256
Source: PLoS One. 2018 Dec 17;13(12):e0208310. doi: 10.1371/journal.pone.0208310 (PMC6296668; doi:10.1371/journal.pone.0208310)
Supplement: S1 Table — (PDF) [file pone.0208310.s001.pdf]

S1 Table. CAP256 gp140 binding ELISA end point titres of individual rabbits

Rabbit 1986 - PBS group				Rabbit 1989 - AddaVax group				Rabbit 1992 - AlhydroGel group		
week	end titre	fold dilution		week	end titre	fold dilution		week	end titre	fold dilution
0	No ELISA signal			0	No ELISA signal			0	No ELISA signal	
4	0.0062	160.33		4	0.0037	271.55		4	0.0004	2552.0847
8	0.0001	17360.13		8	0.0003	3951.21		8	0.0000	33137.8089
12	0.0002	6586.74		12	0.0006	1753.50		12	0.0001	10208.4585
16	0.0000	175508.74		16	0.0001	12930.21		16	0.0000	59367.8497
20	0.0000	43425.09		20	0.0002	4536.84		20	0.0000	59024.8630
22	Missing			22	0.0000	30997.99		22	0.0000	245491.6802
24	0.0000	650256.97		24	0.0000	20282.17		24	0.0000	135687.0726
Rabbit 1987 - PBS group				Rabbit 1990 - AddaVax group				Rabbit 1993 - AlhydroGel group		
week	end titre	fold dilution		week	end titre	fold dilution		week	end titre	fold dilution
0	No ELISA signal			0	No ELISA signal			0	No ELISA signal	
4	0.0114	87.58		4	0.0032	307.70		4	0.0002	4401.7870
8	0.0006	1800.36		8	0.0002	4415.46		8	0.0000	42171.9324
12	0.0017	573.52		12	0.0004	2231.19		12	0.0001	8997.7659
16	0.0002	4959.98		16	0.0000	34468.91		16	0.0000	181850.9967
20	0.0004	2337.38		20	0.0001	13186.90		20	0.0000	59652.8285
22	0.0001	9013.25		22	0.0000	115831.62		22	0.0000	418570.4527
24	0.0002	5329.39		24	0.0000	57892.58		24	0.0000	255583.2663
Rabbit 1988 - PBS group				Rabbit 1991 - AddaVax group				Rabbit 1994 - AlhydroGel group		
week	end titre	fold dilution		week	end titre	fold dilution		week	end titre	fold dilution
0	No ELISA signal			0	No ELISA signal			0	No ELISA signal	
4	0.0051	196.92		4	0.0053	188.41		4	0.0003	3278.7837
8	0.0002	4483.92		8	0.0003	3274.14		8	0.0000	34192.1425
12	0.0011	871.68		12	0.0008	1226.02		12	0.0001	9826.2129
16	0.0001	10354.90		16	0.0001	10408.68		16	0.0000	99620.9586
20	0.0002	5346.98		20	0.0004	2760.17		20	0.0000	37197.0398
22	0.0000	134775.07		22	0.0001	12472.89		22	0.0000	152937.6139
24	0.0000	87191.63		24	0.0001	7329.51		24	0.0000	66035.0357
Rabbit 1995 - PBS group				Rabbit 1997 - AddaVax group				Rabbit 1999 - AlhydroGel group		
week	end titre	fold dilution		week	end titre	fold dilution		week	end titre	fold dilution
0	No ELISA signal			0	No ELISA signal			0	No ELISA signal	
4	0.0017	599.18		4	0.0013	771.81		4	0.0001	9948.964993
8	0.0001	7597.55		8	0.0002	6195.59		8	0.0000	168925.2977
12	0.0002	5275.98		12	0.0004	2558.92		12	0.0000	40222.98618
16	0.0000	79869.96		16	0.0000	24896.42		16	0.0000	632128.9359
20	0.0000	24604.14		20	0.0001	16983.57		20	0.0000	177267.0734
22	0.0000	173118.29		22	0.0000	146775.44		22	0.0000	1193683.382
24	0.0000	118684.57		24	0.0000	52372.65		24	0.0000	384693.7411
Rabbit 1996 - PBS group				Rabbit 1999 - AddaVax group				Rabbit 2000 - AlhydroGel group		
week	end titre	fold dilution		week	end titre	fold dilution		week	end titre	fold dilution
0	No ELISA signal			0	No ELISA signal			0	No ELISA signal	
4	0.0060	167.95		4	0.0059	169.05		4	0.0002	5183.8627
8	0.0023	438.78		8	0.0002	5826.33		8	0.0000	89648.2401
12	0.0035	281.89		12	0.0004	2814.81		12	0.0000	28562.3776
16	0.0005	2006.75		16	0.0001	14814.28		16	0.0000	334636.9018
20	0.0006	1644.62		20	0.0001	9772.75		20	0.0000	38940.7968
22	0.0002	4584.95		22	0.0000	24899.57		22	0.0000	205311.7459
24	0.0003	3298.09		24	0.0000	20530.37		24	0.0000	121005.7117
