# Supplementary material for: The adjuvant AlhydroGel elicits higher antibody titres than AddaVax when combined with HIV-1 subtype C gp140 from CAP256
Source: PLoS One. 2018 Dec 17;13(12):e0208310. doi: 10.1371/journal.pone.0208310 (PMC6296668; doi:10.1371/journal.pone.0208310)
Supplement: S2 Table — (PDF) [file pone.0208310.s002.pdf]

S2 Table. CAP256 V1V2 loop scaffold binding ELISA end point titres of individual rabbits

PBS group		
week 22		
animal	end titre	fold dilution
RB1986	0.0001	7189.20
RB1987	0.0029	339.66
RB1988	0.0003	3811.68
RB1995	0.0001	10205.26
RB1996	0.0006	1646.50
AddaVax group		
week 22		
animal	end titre	fold dilution
RB1989	0.0003	3928.85
RB1990	0.0008	1331.14
RB1991	0.0170	58.78
RB1997	0.0001	7268.25
RB1998	0.0011	904.08
AlhydroGel group		
week 22		
animal	end titre	fold dilution
RB1992	0.0001	10734.06
RB1993	0.0000	27796.61
RB1994	0.0000	29305.62
RB1999	0.0000	36671.38
RB2000	0.0000	84830.69
